# Paraneoplastic Dermatomyositis in a Case of Gallbladder Signet Ring Cell Carcinoma

**DOI:** 10.7759/cureus.10730

**Published:** 2020-09-30

**Authors:** Aritra Paul, Abheepsita Mukhopadhyay, Sudip Kumar Ghosh, Kaustav Samanta, Subhabrata Das

**Affiliations:** 1 Department of Surgery, Nil Ratan Sircar Medical College and Hospital, Kolkata, IND; 2 Department of Surgery, Institute of Medical Sciences and SUM Hospital, Bhubaneswar, IND; 3 Department of Dermatology, R. G. Kar Medical College and Hospital, Kolkata, IND; 4 Department of Surgery, R. G. Kar Medical College and Hospital, Kolkata, IND

**Keywords:** dermatomyositis, paraneoplastic syndrome, gallbladder carcinoma

## Abstract

Dermatomyositis (DM), as a paraneoplastic presentation, is rare, though the incidence of malignancies in patients with DM is very high. While it has been reported with a wide range of cancers, association with gallbladder carcinoma is uncommon. Furthermore, signet ring cell carcinoma detected in the metastasis presumably from our patient's gallbladder mass is itself a rare and highly malignant variant of gallbladder adenocarcinoma. Thus, this association can be considered to be a very novel finding.

We describe the case of a 64-year-old Indian man who presented with cervical lymph node swelling concurrent with facial rashes, periorbital oedema, nasal regurgitation, dysphagia and weakness in his limbs. The presence of characteristic Gottron’s papules and shawl-like truncal rash on examination led to the diagnosis of DM. Fine needle aspiration cytology (FNAC) of the cervical lymph nodes revealed metastatic signet ring cells, and contrast-enhanced CT (CECT) scan of the abdomen showed a significant gallbladder mass with liver infiltration, thus confirming the underlying association.

## Introduction

Dermatomyositis (DM) is a rare disorder, generally considered an idiopathic, inflammatory myopathy. DM as a paraneoplastic manifestation comprises 8% of all myositis cases [1]. It is most commonly associated with carcinoma of breast, lung, stomach and ovary [2]. The pathogenesis of paraneoplastic DM remains relatively unknown, but there exists significant evidence to support the direct involvement of humoral and cell-mediated immunity. Furthermore, the involvement of tumour antigens in provoking an autoimmune-mediated muscle damage is likely. The incidence of DM as a paraneoplastic syndrome rises significantly with age, and thus, all aged DM patients must undergo a meticulous investigation to rule out any underlying malignancy, particularly as tumour resection may lead to remission of disabling DM symptoms in some cases.

We here present a case demonstrating a very rare association of paraneoplastic DM with gallbladder signet ring cell carcinoma as its underlying cause.

## Case presentation

A 64-year-old man was referred to our surgery outpatient department for evaluation of primary source of metastasis in his cervical lymph nodes, which had been proved cytologically as signet ring cell carcinoma. The cervical swellings had appeared around four months ago, but thorough questioning did not reveal any history suggestive of a primary source elsewhere; history was negative for any lumps apart from the swollen cervical lymph nodes, abdominal pain, gastrointestinal bleeding, jaundice, anorexia, haemoptysis, haematuria, testicular swelling, bone pain or headache. However, the patient insisted that he was troubled by the periorbital swelling, and accompanying facial and truncal rash, occurring approximately over the same period of four months. Furthermore, he also noticed progressive nasal regurgitation, dysphagia and weakness in his limbs.

**Figure 1:**
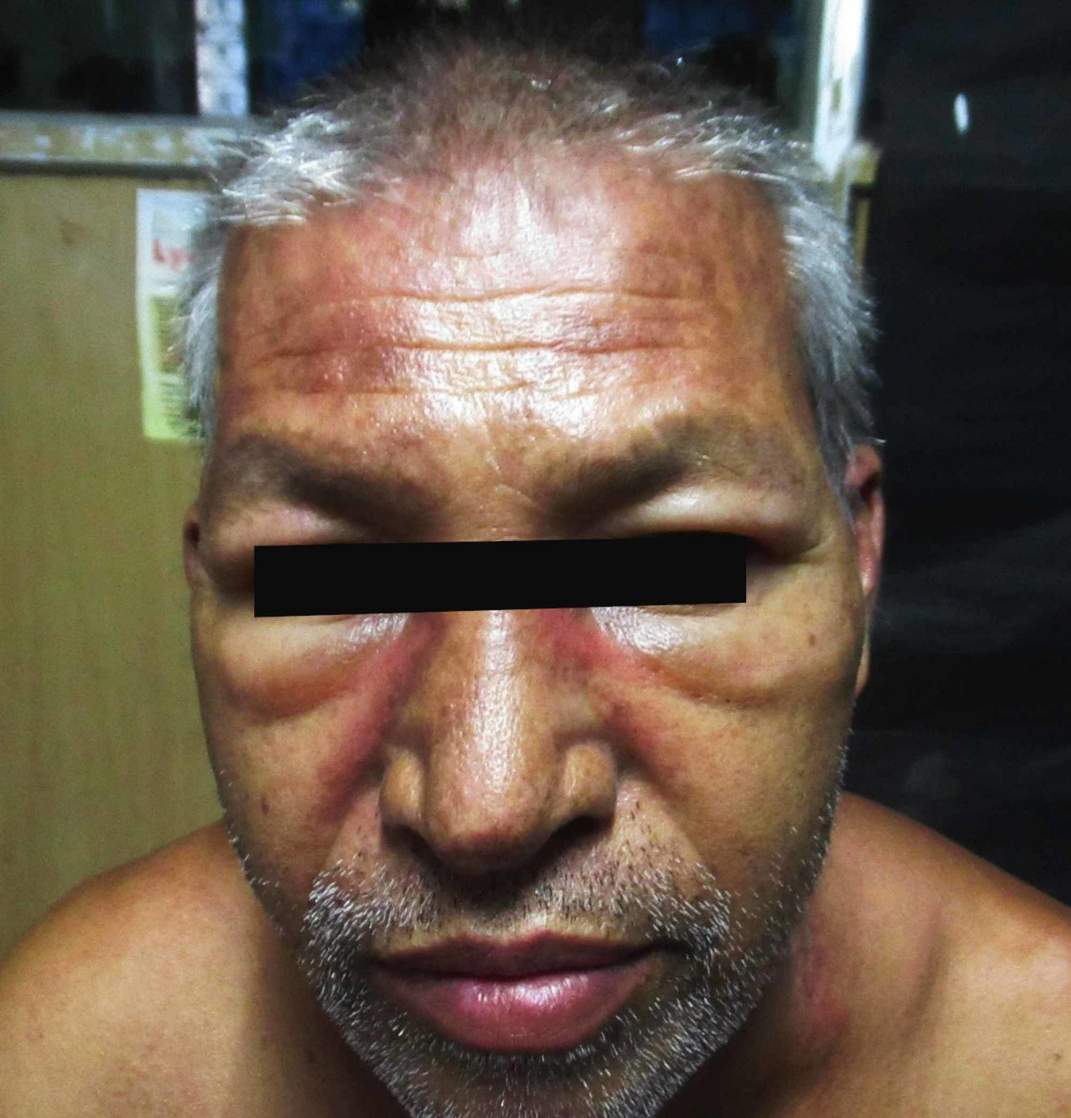
The patient presenting with violaceous periorbital oedema

**Figure 2:**
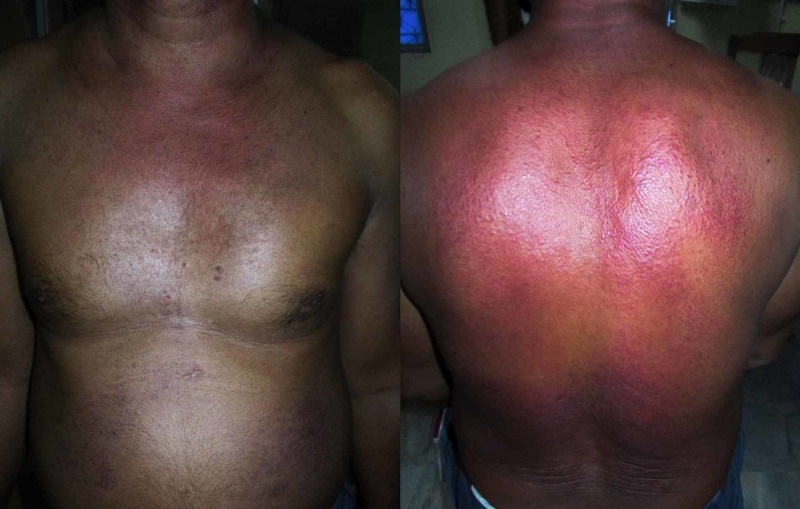
Truncal rash in typical "shawl"-like distribution, characteristic of dermatomyositis (a) Anterior truncal rash. (b) Posterior truncal rash.

**Figure 3:**
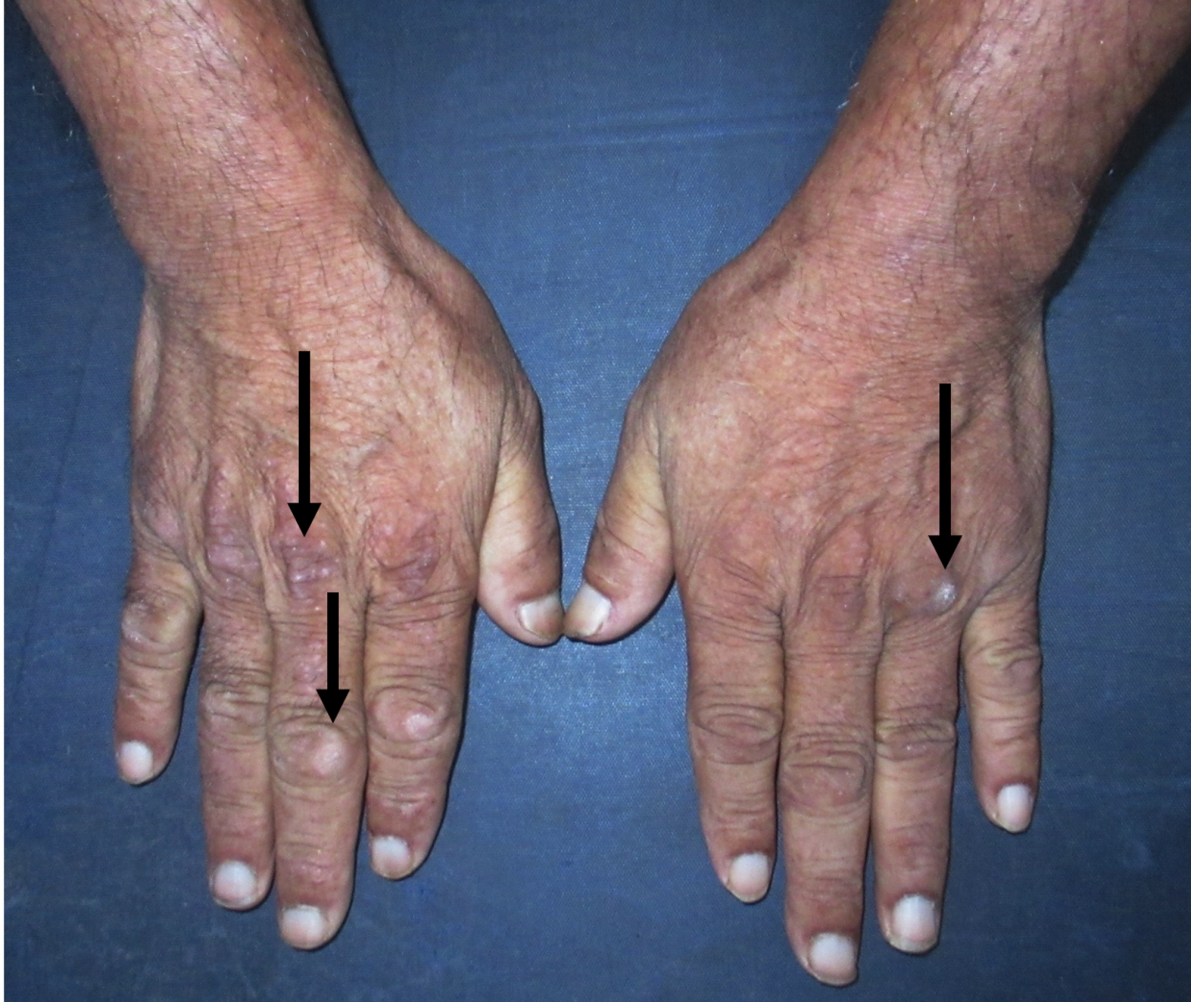
Gottron's papules visible on the knuckles and interphalangeal joints

On examination, several left supraclavicular lymph nodes were enlarged in addition to violaceous periorbital oedema (Figure 1) and rash affecting his nasolabial folds and trunk (Figure 2), along with the presence of papules on his knuckles (Figure 3).

 

 

Consultation with the dermatology department confirmed the diagnosis of "Gottron's papules" on the knuckles and the typical "shawl-like" pattern of truncal rash as characteristic findings of DM.

**Figure 4:**
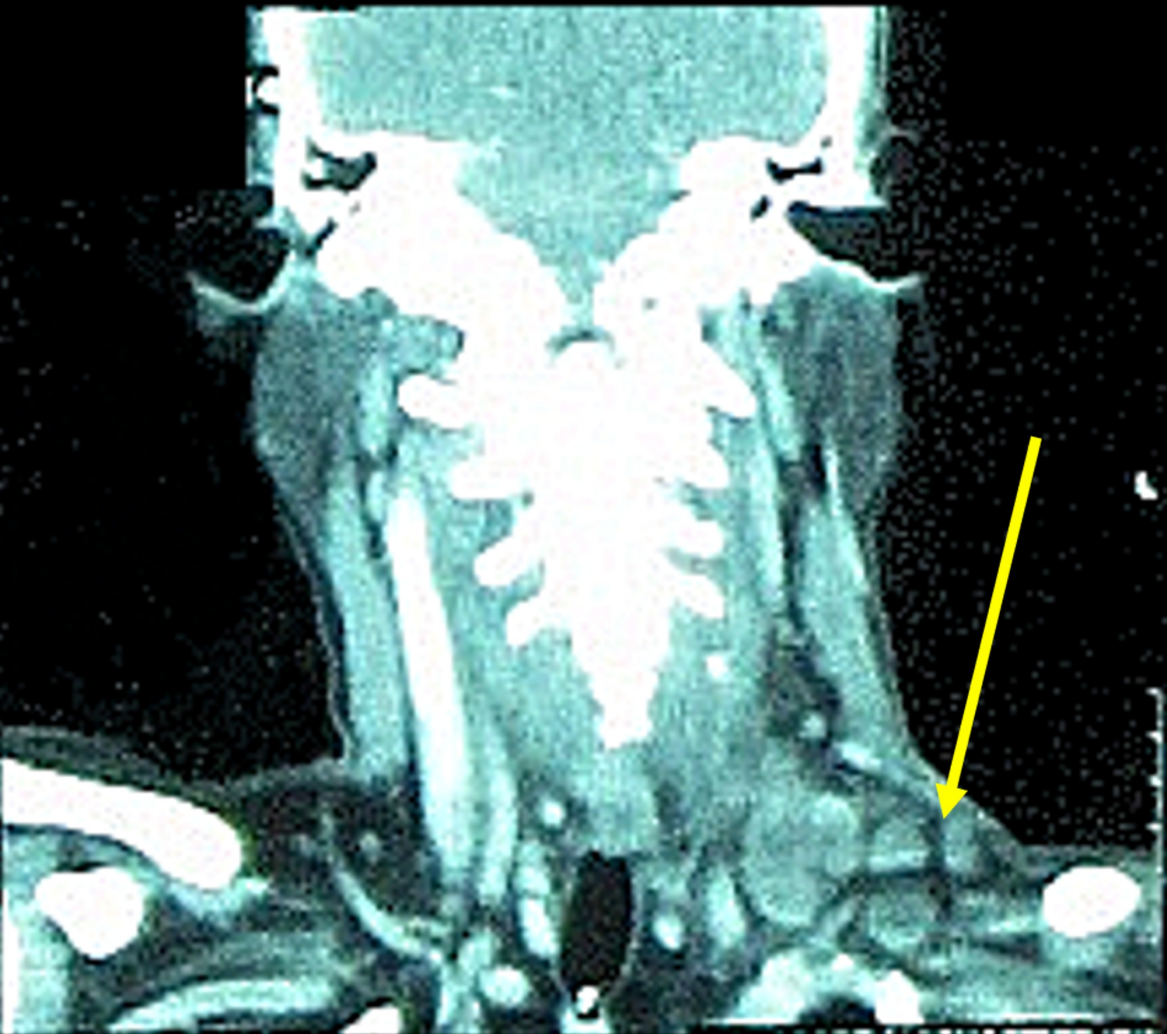
Coronal CT scan of the neck showing cervical lymphadenopathy due to metastatic disease

Fine needle aspiration cytology (FNAC) of the cervical lymph nodes revealed metastatic signet ring cells. Lymphadenopathy was observed on CT scan of the neck (Figure 4).

**Figure 5:**
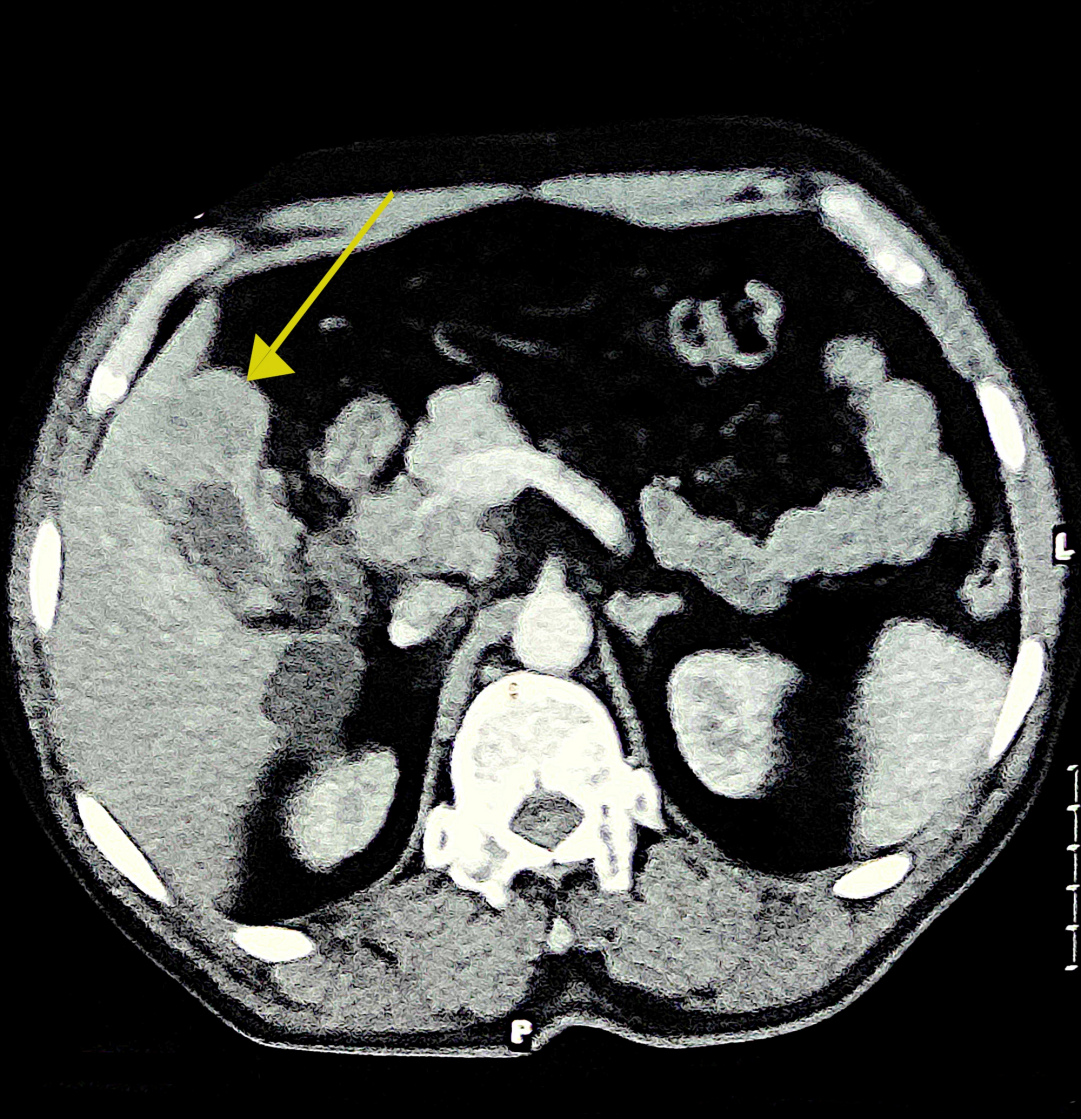
Contrast-enhanced CT scan of the abdomen showing gallbladder mass with infiltration into liver

While the chest X-ray appeared to be normal, contrast-enhanced CT (CECT) scan of the abdomen revealed a significant gallbladder mass with liver infiltration (Figure 5), which is the probable primary source of the cervical lymph node metastases. The case was provisionally diagnosed as paraneoplastic DM due to metastatic gallbladder carcinoma.

## Discussion

DM has an incidence rate of around 9.63 cases per million population, which makes it a very rare disease. It is characterized by a heliotrope rash, proximal muscle weakness, and Gottron’s papules (violaceous, thickened, scaly papules on metacarpophalangeal and interphalangeal joints), and is sometimes accompanied by pitting oedema, dysphagia, nasal regurgitation or aspiration pneumonia and dyspnoea [3]. For up to 40% of patients, skin findings may be the only manifestation [4].

The signet ring cell carcinoma is a highly malignant variant of adenocarcinoma that constitutes of mucin producing cells, and is identified with the typical "signet ring" appearance of epithelial cells on histology. This variety of adenocarcinoma has been found to have a prevalence of 3% among all gallbladder cancers and is a highly aggressive malignancy that portends a poorer prognosis than other adenocarcinomas [5]. It can grow focally or uniformly on the surface, and the latter can often confer a smooth uniform thickening to the gallbladder wall referred to as "linitis plastica". This variant of adenocarcinoma has a similar presentation to its counterparts; however, the malignancy is often already metastatic during the time of diagnosis due to its highly aggressive nature.

The relationship between DM and the occurrence of malignancies has been extensively studied, ever since this relationship was first described back in 1916. Various studies have estimated the risk of cancer in DM through various methods. A retrospective study comprising a cohort of 400 DM patients reported the development of malignancy in 15.8% of patients [6]. Other reports have placed the incidence of DM as a paraneoplastic syndrome in patients with malignancy to be between 6% and 60% [7,8]. The malignancy can occur before, concomitantly, or after the onset of myositis, with a usual peak at two years pre- and post-diagnosis [9]. In our case, a concurrent diagnosis of cancer and DM was made.

Although DM can be associated with a wide range of malignant disorders, its association with carcinoma of the biliary tract is extremely rare, with only a few cases documented globally [1,10-15]. Moreover, there has been only one documented case of paraneoplastic DM in a patient with oesophageal signet ring cell carcinoma [16]. Consequently, our patient seems to have a very rare association of paraneoplastic DM with signet ring cell carcinoma of the gallbladder.

Thus, the intention of reporting this case is to substantiate the possibility of DM as a presentation of signet ring cell carcinoma of the biliary tract, and also to re-emphasize the need for a thorough search for associated malignancy in any case of DM.

## Conclusions

DM, as a paraneoplastic presentation, is rare, though the incidence of malignancies in DM patients is very high. While it has been reported with a wide range of cancers, association with gallbladder carcinoma is quite uncommon. Furthermore, signet ring cell carcinoma detected in the metastasis presumably from our patient’s gallbladder mass is itself is a rare and highly malignant variant of gallbladder adenocarcinoma. Thus, although extremely rare, DM might be associated with signet ring cell carcinoma of the gallbladder, and careful biliary imaging may be indicated, if an underlying malignancy is not evident on clinical examination and other imaging.
